# Association Between Type 1 Diabetes Mellitus and Celiac Disease: Autoimmune Disorders With a Shared Genetic Background

**DOI:** 10.7759/cureus.22912

**Published:** 2022-03-07

**Authors:** Gabriela V Flores Monar, Hamza Islam, Sri Madhurima Puttagunta, Rabia Islam, Sumana Kundu, Surajkumar B Jha, Ana P Rivera, Ibrahim Sange

**Affiliations:** 1 Research, Universidad Central del Ecuador, Quito, ECU; 2 Research, Faisalabad Medical University, Faisalabad, PAK; 3 Research, Dr. Pinnamaneni Siddhartha Institute Medical College, Chinoutpalli, IND; 4 Research, Radha Gobinda Kar (R.G. Kar) Medical College, Kolkata, IND; 5 Research, Jinan University School of Medicine, Guangzhou, CHN; 6 Research, Universidad Americana (UAM) Facultad de Medicina, Managua, NIC; 7 Research, K.J. Somaiya Medical College, Hospital and Research Center, Mumbai, IND

**Keywords:** tissue-transglutaminase antibodies, gluten, celiac disease, hla, type 1 diabetes mellitus

## Abstract

Type 1 diabetes mellitus (T1DM) and celiac disease (CD) are one of the most recognized related autoimmune disorders as they share a common genetic background that has been found in the HLA genotype, more specifically DQ2 and DQ8 molecules. Studies have shown that environmental factors as early or late exposure to cereals in the first months of life or the acquired viral infections have been implicated in the risk of development of autoantigens. CD, in most cases, is asymptomatic; therefore, it goes underdiagnosed. As a result, it has been linked to late consequences as decreased growth, delayed puberty, and anemia. Also, CD has been considered an independent risk factor for nephropathy and retinopathy. Therefore, in T1DM patients, as high-risk individuals, a CD screening has been recommended, especially to analyze their joint management. A gluten-free diet has been studied and linked to possible benefits in glycemic control or decreasing the hypoglycemic episodes in T1DM and preventing in CD the late bowel mucosal damage as gluten has been well documented as the primary trigger of these autoimmune responses. This article has reviewed the concurrent occurrence of T1DM and CD regarding the pathogenesis, clinical overlaps, screening, and management options.

## Introduction and background

Type 1 diabetes mellitus (T1DM) is an autoimmune disorder (AD) characterized by pancreatic beta cell destruction in the islets of Langerhans caused by autoantibodies. This cell destruction leads to insulin deficiency and a consequent state of chronic hyperglycemia [1]. Worldwide, T1DM has an annual increased incidence of approximately 2-3% per year and it has a peak in the group of 10-14 years but can manifest at any age [2]. The incidence of T1DM varies depending on the region, based on genetics and environmental factors, with an incidence of one to three per year in South American and Asian countries, 10-20 per 100,000 in the United States (US) and South European countries [3-5]. T1DM has an identical twin concordance of 30-70%, a sibling risk of 6-7%, and for children whose parents have T1DM, the risk is about 1-9% [6,7].

A considerable proportion of individuals are at a higher risk of T1DM due to its genetic predisposition and variation in their HLA regions. The HLA haplotypes with a stronger correlation are HLA DR4-DQ8 and HLA DR3-DQ2 [8]. T1DM patients are also at a higher risk of developing other ADs. The most common associations are autoimmune thyroiditis and celiac disease (CD). Other related ADs are lupus, rheumatoid arthritis, autoimmune gastritis, Addison's disease, and vitiligo [9]. Approximately 90% of people with a new diagnosis of T1DM have antibodies against B-cell proteins. The main types of autoantibodies that are used as markers are glutamic acid decarboxylase, insulinoma antigen-2, insulin and zinc transporter 8. Individuals with only one antibody do not progress to T1DM but the presence of two or more autoantibodies in children develop in a risk of 84% for a clinical T1DM by the age of 18 [10-12]. The destruction of B cells is not an abrupt process. It is preceded by a prodromal phase that can last approximately 10 years. This stage is characterized by a CD4/CD8 T cell infiltration of the pancreatic islets. In this phase, serum autoimmune markers are positive, but the patient has no symptoms. When the destruction of the islets mass reaches 70-90%, the symptoms of hyperglycemia may be present [13-15]. Children may debut with symptoms of polyuria, weight loss, polydipsia, and diabetic ketoacidosis. However, adults may not manifest common symptoms [16]. Additionally, no single clinical feature can distinguish T1DM from T2DM at diagnosis. Once a high blood glucose level is documented, their classification depends on the correlation of risk factors, clinical features, association with ADs, and biomarkers as autoantibodies [17]. Insulin remains the cornerstone therapy of T1DM, and optimal glycemic control requires multiple insulin regimens that simulate the physiological insulin function. In addition, it is also essential to diagnose and manage additional risk factors and comorbidities to prevent complications [18].

As mentioned above, due to the insidious autoimmune background, patients with T1DM are at an increased risk of developing other ADs, and one of the most relevant is CD. CD has a worldwide frequency of 1%, and it rises at approximately 5% in T1DM patients, which makes it one of the most frequent ADs occurring in T1DM [19]. This review will explore the autoimmune mechanisms, the overlap in clinical features, the importance of early screening, and the adequate joint management of CD and T1DM.

## Review

Shared genetic background and environmental factors

T1DM is an AD in which insulin deficiency results from the destruction of pancreatic beta cells caused by the autoantibodies. This insulin deficiency leads to higher levels of blood glucose [1]. On the other hand, CD is also an AD characterized by its gluten intolerance, which is a protein present in rye, wheat, and barley. When gluten enters the gastrointestinal tract, it reduces into a peptide known as gliadin, which is harmful to celiac patients and predominantly produces gastrointestinal symptoms, mediated by tissue transglutaminase antibodies (tTG), as an Immunoglobulin A (IgA) response [20].

The shared genetic background of T1DM and CD has been well documented based mainly on the presence of the HLA class II genes as DQ2 and DQ8, as they are present in 95% of patients with T1DM and almost 99% of celiac patients (compared to 40% of the unaffected population), representing a significant risk factor for both diseases [21,22]. HLA class II are located in the 6p21 chromosome and have three loci (DR, DQ, and DP). The most involved haplotypes for T1DM are DR and DQ. DQ2 and DQ8 confer a 30-50% risk to develop T1DM. Children with HLA-DR3/DR4 genotype risk of T1DM development is one for each 15-25 versus one in 300 in people in general. In addition, these DBQ1 alleles, such as DQB1*02 and DQB1*03, which enhance the risk to T1DM, are also high-risk factors to develop CD [23,24]. A cross-sectional study conducted by Siddiqui et al. compared the prevalence of HLA-DQ2 and HLA-DQ8 haplotypes in a study population of 175 individuals, including healthy pediatric controls, patients with CD, and patients with CD and T1DM combined. The study showed that the most frequent haplotypes in these populations were DQ2 and DQ8. DQ2 was the only haplotype found in the control individuals in 8.5% of them. In patients with CD, DQ2 was found in 85.7%, DQ8 in 11.4%, and DQ2/DQ8 at the same time in 2.8%. In patients with concurrent CD and T1DM, DQ2 was found in 31.4%, CD8 25%, and DQ2/DQ8 34%, and just nine of these individuals were experiencing CD [20]. In addition to the proven correlation between high-risk factor haplotypes involved in the development of CD and T1DM, the study by Farina et al. showed that more specific than the haplotypes were certain alleles such as HLA DQA1*05; DQB1*02 and HLA DQA1*03; DQB1*03. When DR3-DQA1*05; DQB1*02 are present, they encode for DQ2 molecule and DR4-HLA DQA1*03; DQB1*03 alleles encode DQ8 molecules. These alleles play a significant role in such predisposition to develop the disease [25].

Despite the higher risk of developing both CD and T1DM in genetically predisposed individuals with high-risk HLA genes, it is known that the majority of the population who carry these genes will never present T1DM or CD. Therefore, environmental and external factors are being studied to understand both diseases' predisposition and rising incidence [26,27]. The Diabetes Autoimmunity Study in the Young DAYSI has been continuously studying the causes and risks factors of type 1 diabetes. In one of their investigations, Frederiksen B. et al. reported a period between four and six months of age to introduce cereals without increasing the risks to present islet autoimmunity (IA). Thus, if it was introduced as before as four months or after six months, the risks of developing IA increased. It appears to be the introduction of early exposure of wheat and barley and late exposure of rice and oat. Furthermore, in one of these DAISY studies, it was demonstrated that the risk of developing IA was reduced when gluten was introduced while breastfeeding, independent of the age of exposure to cereals [28]. Ivarsson et al. showed reduced development of CD in children under two years old who were breastfed while the gluten was introduced and probably also reduced its development in the next childhood phase [29]. Additionally, a birth cohort study conducted from 1994 to 2002, with a mean follow-up of four years, studied 1,183 children with increased T1DM risk and determined that when there was a late exposure, the larger amounts of introduced food may be responsible for the increased risk of IA [30]. Furthermore, the Environmental Determinants of Diabetes in the Young (TEDDY) study, a large prospective cohort study of the environmental factors as risks factors for T1DM, during 2004-2009, enrolled 8,676 individuals at three months of age into a 15-year follow-up study. One of their studies, which included 8,676 children, determined the association of respiratory infections and the subsequent risk of developing islet autoantibodies. It also studied their time lapse of seroconversion. The number of infections occurring during the first nine months of age was related to a statistical increase in IA risk, with a hazard of IA that increased approximately 5.6% for every one per year rate increase in infections from the respiratory tract. The leading respiratory infections linked independently with IA were an influenza-like disease, sinusitis, common cold, and laryngitis or tracheitis [31].

The summary of studies related to shared genetic background and environmental factors of T1DM and CD are shown in Table 1.

**Table 1:** Summary of mentioned articles about shared genetic background and environmental factors in T1DM and CD T1DM, type 1 diabetes mellitus; CD, celiac disease; HR, hazard ratio; OR, odds ratio; HLA, human leukocyte antigen; US, United States; IA, islet autoimmunity

Author	Year	Design	Population	Global Sample	Results	Conclusions
Siddiqui et al. [20]	2021	Cross-sectional	Pediatric samples from the Isra University Hospital, Liaquat University of Medical and Health Sciences Hospital and Asian Institute of Medical Sciences Hospital, Hyderabad, India	175 pediatric individuals with T1DM or CD	In patients with CD, DQ2 was found in 85.7%, DQ8 in 11.4%, and DQ2/DQ8 at the same time in 2.8%. In the group of concurrent CD and T1DM, DQ2 was found in 31.4%, CD8 in 25%, and DQ2/DQ8 in 34% and just nine of these individuals were experimenting CD	The most frequent haplotypes present in these population were DQ2 and DQ8 coming after and they could be used for a more accurate diagnosis in selected cases
Farina et al. [25]	2019		Children enrolled in a Hospital of Naples, Italy	21 patients with T1DM	DR3-DQA1*05 and DQB1*02 alleles encode for DQ2 molecule and DR4-HLA DQA1*03 and DQB1*03 alleles encode DQ8 molecules	DQ2.5 and DQ8 risk alleles are more frequent than non-associated alleles in T1DM patients
Frederiksen et al. [28]	2013	Longitudinal, observational study	Newborns at St. Joseph’s Hospital in Denver, Colorado	1,835 children at risk for T1DM	Early exposure to any solid food (HR: 1.91. 95%. CI: 1.04-3.51) and late first exposure (HR: 3.02; 95% CI: 1.26-7.24) predict risk of development of T1DM	There is a period of time, between four and six months, to introduce solid foods without increasing the IA risks. The risk of developing IA was reduced when gluten was introduced while breastfeeding
Ivarsson et al. [29]	2002	Prospective study	Swedish children	524 children	The risk to develop CD was reduced if a child is being breastfed when gluten is introduced in its diet (OR: 0.59; 95% CI: 0.42-0.83)	Reduced development of CD in children under two years old who were breastfed while the gluten was introduced and probably also reduced risk of development in the next childhood phase
Norris et al. [30]	2003	Birth cohort study	Newborns at St Joseph's Hospital in Denver, Colorado	1,183 children at increased type 1 DM risk	Children who were exposed to cereal in the first three months of age (HR: 4.32; 95% CI: 2.0-9.35) and the ones who were exposed after six months (HR: 5.36; 95% CI: 2.08-13.8) had an increased risk of IA	The risk of IA increases if exposure to cereals is outside a window of time between four and six months of age
Lönnrot et al. [31]	2017	Prospective international cohort study	Children enrolled in six clinical research centers: three in the US (Colorado, Georgia/Florida, and Washington State) and three in Europe (Finland, Germany, and Sweden)	8,676 children	The risk of IA was associated with the number of respiratory infections given in a nine-month frame of time (p < 0.001). The hazard of IA increased approximately 5.6% for every one per year rate increase in infections from the respiratory tract	Respiratory infections occurring in young children were associated with the subsequent risk of autoimmunity

Clinical implications and impact on quality of life

A systematic review was conducted from 1990 to 2015 to estimate the global prevalence and impact of diabetes for 2015 and 2040. The findings reported that in 2015 there were 415 million people (estimated between 340 and 536 million) between 20 and 79 years with a diabetes diagnosis. It was also found that five million deaths were attributed to diabetes, representing an estimated global health expense of 673 billion dollars. Most patients lived in low- and middle-income countries representing 75% of the diagnosed people. It is predicted that by 2040 these numbers will rise to an estimated 642 million people with diabetes, contributing to worse financial, social, and health implications all around the globe [32]. More specifically, talking about diabetes correlation with CD, the first study of its kind was conducted by Walker-Smith J in 1969. Since then, some studies have been developed to get an insight into its epidemiology, clinical implications, and long-term consequences [33]. Nowadays, CD and T1DM have an approximate worldwide frequency of 1% and 0.5%, respectively. A study conducted by Abid et al. reported a rise of approximately 15.4% in serologically proven CD prevalence and 6.9% in biopsy-proven celiac disease (BPCD) prevalence in T1DM patients [34,35].

CD must be taken into account if faced with any suspicious characteristics. CD's suggestive and main gastrointestinal symptoms associated with T1DM are diarrhea (which affects almost 50% of patients), flatus, weight loss, and altered bowel habits. Additional symptoms include neuropathy, ataxia, and constipation, among a variety of others. Its clinical signs include iron deficiency anemia and decreased low bone density as the most studied. CD may also develop non-classical symptoms or be asymptomatic. Those individuals with silent CD were characterized by having seropositive tests without manifestations of any kind [36,37].

One of the most significant studies of the prevalence of CD in T1DM studied the data of 52,721 youth with T1DM in three continents: the US, Australia, and Europe (more precisely, United Kingdom, Germany, and Austria). BPCD was evidenced in 1,835 individuals, representing 3.5%, and the median age at diagnosis was 8.1 years (5.3-11.2 years). CD was diagnosed less than a year after T1DM diagnosis in 35%, between one and two years in 18%, three and five years in 23%, and more than five years in 17%. The prevalence of CD went from 1.9% in US data to 7.7% in Australia and was statistically significantly higher (p < 0.001) in females than males (4.3% vs 2.7%). T1DM individuals with coexisting CD were younger at the diagnosis than the ones with T1DM alone (5.4 years vs 7 years). Height standard deviation score was lower in those with accompanying CD. Hemoglobin A1c (HbA1c) did not show any significant difference [38].

The concomitant presence of these disorders leads to an increased rate of complications arising from these disorders, which highlights the importance of screening for such comorbidities. A large multicenter longitudinal analysis from German Australian Patienten Verlausdokumentation (DPV) database also was performed and consisted in 56,514 patients with diabetes duration less than 20 years, divided into three categories: No CD (50,933 patients), biopsy-confirmed CD (812 patients), and clinically suspected or positive antibodies CD (4.769 patients). Nephropathy and retinopathy were present earlier in those with concomitant T1DM and CD. Nephropathy analyzed as microalbuminuria was present almost 10 years earlier in patients with CD versus non-CD: 32.8 years (29.7-42.5) vs 42.4 years (41.4-43.3). In 25% of patients, retinopathy presented at 26.7 years (23.7-30.2) in CD patients versus 33.7 in non-CD patients. Thus, evidence showed that the presence of CD in a T1DM patient represents an independent risk factor for nephropathy and retinopathy. In consequence, the study recommended serologic tests of CD even in asymptomatic T1DM patients [39]. A study that also collected their data from the multicenter longitudinal analysis previously mentioned (DPV) analyzed the association between depression in children and young adults with T1DM and CD. Data included four categories of patients: 73,699 with T1DM only, 3,379 participants with both T1DM and CD, 1,877 participants with T1DM and depression, and 112 participants with all three diagnoses: T1DM, CD, and depression. It was concluded that depression was more frequent in T1DM with concomitant CD patients, with higher HbA1c and anxiety and eating disorders than the T1DM group only. Thus, screening for depression was recommended as routine to improve results and quality of life in those patients [40]. Baddada et al. carried out a retrospective study in India, with records of 109 patients with CD under the age of 20 years during April 2008 and March 2013. The study divided the individuals into two groups: CD alone (78.9%) and concurrent T1DM and CD (21.1%) patients. It was demonstrated that patients with CD alone had a more delayed diagnosis than those with accompanying CD and T1DM. Consequently, it led to more anemia, short stature, and delayed puberty [41].

Studies related to the clinical implications and impact on quality of life are depicted in Table 2.

**Table 2:** Summary of mentioned articles about clinical implications of T1DM and CD and impact on the quality of life T1DM, type 1 diabetes mellitus; CD, celiac disease; BPCD, biopsy-proven celiac disease; anti-tTG, anti-tissue transglutaminase antibodies; US, United States; HbA1c, hemoglobin A1c; GFD, gluten-free diet

Author	Year	Design	Population	Global Sample	Results	Conclusions
Abid et al. [35]	2011	Longitudinal study	T1DM children in the Royal Belfast Hospital for Sick Children in United Kingdom	468 children with T1DM	Mean age at T1DM diagnosis was 6.8 years and CD diagnosis 11.1 years. The majority (10 out of 11) had improvement in their gastrointestinal symptoms and six out of eight did not present more high-risk hypoglycemic episodes. However, the daily insulin requirement went up, from 0.88 to 1.1 unit/kg/day	A GFD improved the gastrointestinal symptoms and decreased episodes of severe hypoglycemia but the insulin requirement increased
Craig et al. [38]	2017		Youth with T1DM in three continents: US, Australia, and Europe (more specifically United Kingdom, Germany, and Austria)	52,721 individuals <18 years of age	BPCD was evidenced in 1,835 individuals, which represent 3.5% and the median age at diagnosis was 8.1 years (5.3-11.2 years). CD was diagnosed less than a year after T1DM diagnosis in 35%, between one and two years in 18%, between three and five years in 23%, and more than five years in 17%. The prevalence of CD went from 1.9% in US data to 7.7% in Australia and was higher in females than males (4.3% vs 2.7%).	T1DM individuals with coexisting CD were younger at the diagnosis than the ones with T1DM alone. HbA1c did not show any significant difference but height standard deviation score was lower in those with concomitant CD; therefore, a follow-up is recommended.
Rohrer et al. [39]	2015		Patients with T1DM from the German-Austrian DPV Database	56,514 individuals with T1DM less than 20-year duration	Nephropathy analyzed as microalbuminuria presented almost 10 years earlier in patients with CD versus non-CD: 32.8 years (29.7-42.5) vs. 42.4 years (41.4-43.3), and retinopathy in 25% of patients presented at 26.7 years (23.7-30.2) in CD patients versus 33.7 in non-CD patients	The presence of CD in a T1DM patient represents an independent risk factor for nephropathy and retinopathy, so in consequence the study recommends serologic tests of CD even in asymptomatic T1DM patients
Tittel et al. [40]	2021		Children and young adults from the German-Austrian DPV Database	79,067 children, adolescents, and young adults	In T1DM + depression, HbA1c was higher (9.0% [8.9-9.0]). in CD + T1D + depression (8.9% [8.6-9.2]), compared with T1D only (8.2% [8.2-8.2]). Also, anxiety, schizophrenia, and eating disorders are more frequently found in the T1D + CD + depression group compared with T1D group (p < 0.001).	Depression is significantly more frequent in T1DM with concomitant CD patients, also along with a higher HbA1c and anxiety and eating disorders compared to the T1DM group only. Thus, a screening for depression is recommended as routine as well to improve results and quality of life in those patients
Bhadada et al. [41]	2017	Retrospective study	Patients under 20 years old from Chandigarh, India	109 patients with T1DM under 20 years old	The age at diagnosis of CD and the time frame between the diagnosis of T1DM and CD were 11.5 ± 4.6 versus 13.8 ± 3.4 years and 48.8 ± 43.3 versus 20.2 ± 31.8 months in groups of CD alone and CD plus T1DM, respectively. Short stature (87% vs. 40.9%), anemia (80.9% vs. 45%), and delayed puberty (61.9% vs. 29.4%) were more frequent in CD alone group.	Patients with CD alone had a more delayed diagnosis than the ones with concurrent CD and T1DM and consequently to its late diagnosis, it led to more incidence of anemia, short stature, and delayed puberty

Early screening and diagnosis of CD in T1DM patients

Screening patients of CD has been controversial since most are asymptomatic, and the long-term consequences depend on the patients' characteristics and risk factors. A cohort study performed between 1995 and 2015 included individuals diagnosed with T1DM between one and 35 years old with no previous CD diagnosis, using The Health Improvement Network, a database of more than 13 million people from primary care in the United Kingdom. A greater risk of developing CD was found in patients diagnosed with T1DM at a younger age and in the female sex. Even though CD could develop at any age after T1DM, it was recommended that CD screening should be done in childhood and adulthood [42]. According to the American Diabetes Association, an early screening for CD in T1DM-diagnosed children is recommended, the earlier, as most CD diagnoses are being made within the first years of T1DM diagnosis. Thus, in this case, as soon as T1DM is diagnosed, screening for CD should be made by measuring IgA tTG and proven serum IgA levels within the normal range. Immunoglobulin G (IgG) tTG or IgG deamidated gliadin antibodies should be measured if IgA levels were deficient. After this first approach, a second screening should be done within two years of T1DM diagnosis and again after five years. A more frequent screening should be made if a child had a first-degree relative with CD or presented CD-related symptoms. Among these symptoms, the tendency of growth and weight gain are included [43]. In order to confirm the diagnosis, a bowel biopsy if the antibody testing is positive is recommended [44]. European guidelines suggest that in children with high antibody titers (10 times greater than the normal) and symptoms, the biopsy to confirm the diagnosis may not be necessary. However, they do not affirm that this approach could also be adopted for asymptomatic children [45].

Nonetheless, data from the prospective Swedish study from 2005 to 2010 were collected, and 2,035 children and adolescents with T1DM were included. In 60 children with anti-tissue transglutaminase antibody (anti-tTG) greater than 10 times, their CD was confirmed in their biopsies and correlated with the bowel mucosal damage using the Marsh classification. Therefore, it was recommended that the levels of anti-tTG in children and adolescents could be used as a noninvasive diagnostic method [42]. 

On the other hand, referring to the adult population with T1DM, it is recommended that they undergo CD screening if they present related symptoms, signs, or laboratory findings. The method used for screening is still the measurement of anti-tTG [42]. A study was conducted in Finland with a cohort of 520 children between 0 and 17 years old with BPCD, which were divided into two groups depending on CD detected at T1DM surveillance by serological screening and the ones diagnosed with CD because of clinical suspicion. It was proven that in the serological screening group, the patients were less affected by clinical symptoms and decreased growth than the ones diagnosed after clinical suspicion. Also, both groups showed signs of malabsorption and similar advanced intestinal mucosal damage. They concluded that screening for CD should be done in every patient with T1DM. Thus, it is essential to keep these screening and diagnostic protocols in mind when dealing with such clinical scenarios [46].

Joint management

It is important to acknowledge that in previously diagnosed T1DM individuals, the outcome would be improved if there is combined management of their comorbidities. In this particular case, when CD is diagnosed, focus mainly on whether or not a gluten-free diet (GFD) is followed. One retrospective study with the data of 779 patients with T1DM, from which 668 had an anti-tTG IgA test, was followed from 2009 to 2019 to study the frequency of spontaneous normalization of serology tests for CD and the frequency of BPCD in T1DM patients. Positive serologic tests were detected in 103 T1DM patients (15.4%). The majority of CD cases (76.1%) were found at the diagnosis of T1DM and 21.7% in the first five years. In conclusion, 97.8% of cases were diagnosed in the first five years of T1DM and 2.2% in the following years. However, the percentage of BPCD was just 6.9% at the time of the diagnosis. Additionally, in 23.3%, the positive autoantibodies spontaneously normalized without a GFD. Therefore, this study recommends the serological test for diagnosis and follow-up instead of biopsy-required CD diagnosis. They also suggested not initiating an immediate GFD therapy in asymptomatic patients or with a mild autoantibodies title test because it adds an unnecessary burden on the diagnosed children and their families [47]. 

An open randomized controlled trial in 2019, developed by Kaur et al., was the first study of its kind evaluating the GFD for CD in T1DM. In this study, they divided the patients into two groups: those who received a GFD from those on a regular diet for a year and studied the frequency of hypoglycemia and the effects on height, weight, HbA1c, insulin dose requirement, and bone mineral homeostasis as outcomes. The number of hypoglycemic episodes per month declined in patients under a GFD (3.5 episodes at the beginning of the study versus 2.3 episodes at the sixth month). In addition, the HbA1c was reduced by 0.73% in the GFD patients and elevated by 0.99% in the standard diet [48]. The previously mentioned study performed by Abid et al., to observe the metabolic and clinical effects of a GFD in T1DM-diagnosed children with proved CD, collected data between 2000 and 2007 in 468 children, and 11 of them were diagnosed with CD. They were analyzed before and after a 12-month GFD, and in the results, the mean age at T1DM diagnosis was 6.8 years and CD diagnosis 11.1 years. The majority (10 out of 11) had improvement in their gastrointestinal symptoms, and six out of eight did not present more high-risk hypoglycemic episodes. However, the daily insulin requirement went up, from 0.88 to 1.1 unit/kg/day [35]. At the moment, the benefits of dietary intervention in T1DM patients with CD are not clear. A prospective multicenter, randomized controlled study, including patients with T1DM and CD, between eight and 45 years, has been ongoing since 2012 in Ontario, Canada. This study will evaluate the results on HbA1c in patients who follow a GFD and those on a gluten-containing diet for one year. They will also measure other outcomes as bone mineral density and quality of life [49].

Studies related to screening, diagnosis, and joint management of T1DM and CD are depicted in Table 3.

**Table 3:** Summary of mentioned articles about screening, diagnosis, and joint management of T1DM and CD T1DM, type 1 diabetes mellitus; CD, celiac disease; BPCD, biopsy-proven celiac disease; anti-tTG, anti-tissue transglutaminase antibodies; HR, hazard ratio; US, United States; HbA1c, hemoglobin A1c; CI, confidence interval; GFD, gluten-free diet

Author	Year	Design	Population	Global Sample	Results	Conclusions
Vajravelu et al. [42]	2018	Cohort Study	Individuals from United Kingdom primary care database	9,180 patients diagnosed with T1DM between one and 35 years old with no previous diagnosis of CD	CD was diagnosed in 196 T1DM patients (2%) during the study. A younger age at T1DM at diagnosis (HR 0.91 [95% CI 0.88-0.94]) and female sex (HR 3.19 [95% CI 1.39-7.34]) were associated with an increased risk of CD.	The greater risk of developing CD was found in those who were diagnosed of T1DM at a younger age and in the female sex. Even though CD could develop in any age after T1DM, CD screening should be done in childhood and adulthood
Paul et al. [45]	2018	Prospective study	Children and adolescents at the Bristol Royal Hospital in England	2,035 children and adolescents with T1DM	157 T1DM children with no clinical symptoms were diagnosed with CD. 53.5% had anti-tTG >10× ULN (normal <10 IU/mL) and 89% were from high-risk groups; all of this percentage had a positive histological evidence of small bowel enteropathy	In children and adolescents, the levels of anti-tTG could be used as a noninvasive diagnostic method
Laitinen et al. [46]	2017	Cohort study	Children and adolescents at the Tampere University Hospital, Finland	520 children and adolescents between 0 and 17 years	Children from the screening had less decreased growth (p = 0.016) and symptomatology (p < 0.001) at diagnosis than the children that were tested after they had symptoms.	In the serological CD screening group, the patients were less affected by clinical symptoms and decreased growth than the ones diagnosed after clinical suspicions. Also, both groups showed signs of malabsorption and similar advanced intestine mucosal damage. Therefore, a screening for celiac disease should be done in every patient with T1DM.
Unal et al. [47]	2021	Retrospective study	T1DM at the University of Health Sciences in Turkey	779 T1DM patients	The majority of CD cases (76.1%) were found at the diagnosis of T1DM and (21.7%) in the first five years, making together the 97.8% of cases diagnosed in the first five years of T1DM diagnosis; and the rest of the cases (2.2%) in the following years. However, the percentage of BPCD were just 6.9% at the time of the diagnosis. Additionally, in 23.3% cases, the positive autoantibodies spontaneously normalized without a GFD.	A serological test for diagnosis and follow-up is recommended, instead of biopsy-required diagnosis of CD. Also, not initiating an immediate GFD therapy is suggested, especially in asymptomatic patients or with a mild value of autoantibodies test because it adds an additional burden in the diagnosed children and their families.
Kaur et al. [48]	2019	Randomized controlled trial	Patients with T1DM from India	320 patients with T1DM	The number of hypoglycemic episodes per month declined in the patients under a GFD (3.5 episodes at the beginning of the study versus 2.3 episodes at the sixth month). Also, the HbA1c was reduced by 0.73% in the GFD patients and elevated in 0.99% in the normal diet.	In patients with T1DM and CD, following a GFD could decrease the hypoglycemic episodes and could lead to an improved glycemic control.

Limitations

In this review, we brought together the main linked characteristics of T1DM and CD, beginning with its genetic and environmental background to improve the understanding of its foundation and, therefore, implying better handling of its diagnosis, screening, and management. Despite displaying all these evidence-linked features all together in a sole study, we also found some limitations. First, our research was done mainly in one database (PubMed). Second, different study models were collected with different samples and populations, so there is no strict homogeneity in the review. Finally, further studies about the correct CD screening and follow-up in T1DM patients are necessary. Also, research about the short- and long-term effects of a GFD is needed because evidence regarding this topic is lacking in the literature.

## Conclusions

The shared genetic background of T1DM and CD has been established mainly on the HLA class II genes as DQ2 and DQ8 as they are present in 95% of patients with T1DM and 98% of CD patients. Nevertheless, this genetic predisposition alone is not sufficient for the disease to appear. It has been studied that the time frame in which food is introduced in the first months of life determine the consequent risks along with viral infections and the role of breastfeeding as a protective factor. The importance of an early CD screening in T1DM patients is based on the possible consequences of an underdiagnosed concomitant CD, as decreased growth and low bone density and anemia. Also, in the long run, the risk of developing vascular complications as nephropathy and retinopathy is an independent risk factor in patients previously diagnosed with T1DM. Therefore, early screening is recommended by measuring mainly IgA tTG, with a close follow-up, independent of age, as CD could develop at any time frame after T1DM's first diagnosis. The subsequent management of CD and T1DM with a GFD has been controversial as it has been proven that autoantibodies could spontaneously normalize without a GFD. However, if they remain positive, a GFD could reduce the early CD symptoms and the number of hypoglycemic episodes in T1DM patients. Thus, additional studies are needed, especially on the benefits of possible future treatments where the benefits outweigh the risks.
